# A New QTL for Plant Height in Barley (*Hordeum vulgare* L.) Showing No Negative Effects on Grain Yield

**DOI:** 10.1371/journal.pone.0090144

**Published:** 2014-02-28

**Authors:** Junmei Wang, Jianming Yang, Qiaojun Jia, Jinghuan Zhu, Yi Shang, Wei Hua, Meixue Zhou

**Affiliations:** 1 Institute of Crop and Nuclear Technology Utilization, Zhejiang Academy of Agricultural Sciences, Hangzhou, Zhejiang, PR China; 2 Tasmanian Institute of Agriculture and School of Land and Food, University of Tasmania, Kings Meadows, Tasmania, Australia; Department of Agriculture and Food Western Australia, Australia

## Abstract

**Introduction:**

Reducing plant height has played an important role in improving crop yields. The success of a breeding program relies on the source of dwarfing genes. For a dwarfing or semi-dwarfing gene to be successfully used in a breeding program, the gene should have minimal negative effects on yield and perform consistently in different environments.

**Methods:**

In this study, 182 doubled haploid lines, generated from a cross between TX9425 and Naso Nijo, were grown in six different environments to identify quantitative trait loci (QTL) controlling plant height and investigate QTL × environments interaction.

**Results:**

A QTL for plant was identified on 7H. This QTL showed no significant effects on other agronomic traits and yield components and consistently expressed in the six environments. A sufficient allelic effect makes it possible for this QTL to be successfully used in breeding programs.

## Introduction

Most agronomically and economically important traits are controlled by quantitative trait loci (QTL), and are known to be affected by environmental factors. Modern molecular techniques and recent advances in QTL analysis methods enable QTL controlling complex traits to be mapped and elucidated [1].

Plant height is influenced by many qualitative genes and QTL [2]. In barley, plant height is controlled by dwarfing, semi-dwarfing, and other plant height genes. Dwarfing genes are not useful in barley breeding due to depressing vigour and grain yield. Semi-dwarfing genes are useful and more common than dwarfing genes [3], [4]. These semi-dwarfing genes, including semi-brachytic 1 (*uzu1*) [5], semi-dwarf 1 (*sdw1* or *denso*) [6], breviaristatum- e (*ari-e*) [7], and short culm 1 (*hcm1*) [8], are mainly used in barley improvement. The *uzu1*, *sdw1 and denso* genes are located on chromosome 3H [5], [9] and are very close to each other [10] with the *sdw1* gene being allelic to *denso* [11]. The *ari-e* gene is located on chromosome 5HL [7] and the *hcm1* is located on chromosome 2HL [12]. Additional semi-dwarfing genes have been found in landraces and cultivars, but some have not been localized in the genome yet [3], [4]. More recently, a QTL on chromosome 7H were reported to be responsible to a semi-dwarfing gene [13].

Grain yield is generally controlled by many genes and can be dissected into series of component parts including spike number, kernel number, kernel weight and thousand-kernel weight. Adaption to environment through heading date can maximise yield potential and decreased plant height was used to reduce yield loss arising from lodging and to increase the harvest index [14]. In East Asia and Europe, the short-stature cultivars with *uzu1* or *denso* gene have been developed by barley breeders widely to reduce lodging and increase grain yield [5], [11]. The *sdw1* gene, which has been shown to be allelic to the *denso*, has been widely used to develop feed barley cultivars in western USA, Canada, and Australia [15]. However, these semi-dwarfing genes were found to be linked with some unfavourable traits [10], [16], [17]. There are also relationships between heading date and plant height in barley, in which apical growth is terminated by flowering [18]. Three alleles at the *sdw1* locus were found to delay heading [11] and some are temperature and/or day length sensitive [10].

In our previous studies, semi-dwarfing genes were identified in a Chinese landrace variety, TX9425, which are in a similar position of *uzu1* gene and showed tight linkage with spike morphology, including grain density, spike length and awn length [10], [16]. More interestingly, plant height of this variety varies widely with different growth conditions and segregations were found between TX9425 and another semi-dwarf variety Naso Nijo. To investigate whether any new QTL for plant height exist and if these QTL have any effects on other agronomic traits and yield components, 182 DH lines from this cross were grown under six different environments (2 years x 3 sites) to identify new QTL and to investigate the interaction between environments and QTL for plant height and other traits.

## Materials and Methods

All the field trials were conducted in experimental farms with no concerns with protection of wildlife, etc. No specific permissions were required for these locations/activities since these areas were designed for experimental trials. The field studies did not involve endangered or protected species.

### Plant material

A population of more than 300 doubled haploid (DH) lines was produced from the F_1_ of the barley cross between TX9425 and Naso Nijo by the anther culture method. TX9425 is a Chinese two-rowed feed variety, which showed low malting quality [19] but good tolerance to various stresses [19]–[21]. In contrast, Naso Nijo is a Japanese two-rowed malting barley with good agronomic traits and less tolerance to various stresses. Among all the DH lines, only about 10% showed “TX9425 type spike morphology” (short awn and high grain density) which is co-segregated with *uzu* dwarf gene [16]. This is due to a segregation distortion in the process of anther culture, which is a common phenomenon in plant [22]. A total of 188 lines were genotyped with DArT and SSR markers. Since *uzu* gene showed close linkage with many other traits [10], [16], only 6 lines with “TX9425 type spike morphology” were included in genotyping and they were all excluded from further QTL analysis.

### Experimental design

The DH lines and parents were grown in six different environments with different ecological conditions. These included Hangzhou (HZ) of Zhejiang province, Yanchen (YC) of Jiangsu province, and Baoshan (BS) of Yunnan province, in two successive growing seasons, 2006–2007 and 2007–2008. All trials were sown between late October and early November. In field trials, 150 seeds of each line were grown in a 2-m row with in 0.25 m between rows. All agronomic managements, including fertilization, weed and disease control, were in accordance with local practice. All experiments were arranged as a randomized complete block design with three replications. At maturity, all grains of each line were harvested for further analysis.

### Phenotypic evaluation of agronomic Traits

Plant height (PH), heading date (HD), spike length (SL), awn length (AL), grain number per spike (GN), 1000-kernel weight (KW) and grain yield per plot (GY) were evaluated. The recorded traits and methods of measurement are listed in Table 1.

**Table 1:** List of agronomic traits investigated in six environments.

Abbreviation	Trait	Method of measurement	Unit
**HD**	**Heading date**	**Number of days from sowing to the time when in 50% ear had emerged from the flag leaf sheath**	**d**
**PH**	**Plant height**	**Plant height measured from soil surface to tip of spike (excluding awns)**	**cm**
**SL**	**Spike length**	**Length from the base of spike to the tip of the terminal spikelet (excluding awns)**	**cm**
**AL**	**Awn length**	**Length of awn in the central spikelet**	**cm**
**GN**	**Grain number**	**Number of grains per spike**	**No**
**KW**	**Kernel weight**	**Average weight of 1000 kernels**	**g**
**GY**	**Grain yield**	**Weight of the grain harvested from the whole line and dried for 1**–**2 days**	**g**

### Map construction

Leaves from three-week-old seedlings (a single seedling per genotype) were harvested and genomic DNA was extracted according to a modified CTAB method described by Stein et al. [23]. 326 SSR markers were selected after searching the web site (http://www.genetics.org/cgi/content/full/156/4/1997/DC1). SSR marker genotyping was conducted by the procedure of Ramsey et al. [24]. These primers were used to screen for polymorphisms between the parental varieties Naso Najo and TX9425. Only primers with clear polymorphisms were used to genotype the DH population. Genomic representations and preparation of the “discovery arrays” and “polymorphism-enriched arrays” for DArT analysis were as explained by Wenzl et al [25]. A quality parameter Q, which is the variance of the hybridization intensity between allelic states as a percentage of the total variance, was calculated for each marker. Only markers with a Q and call rate both greater than 80% were selected for linkage analysis. After removing non-polymorphic and low quality markers, 551 DArT markers and 75 SSR markers were used for map construction. Software package JoinMap 4.0 [26] was used to construct a complete linkage map. The map was finally compared with two DArT consensus maps [27], [28] and 7 markers in the new map which were located on different linkage groups in the consensus maps were added an “a” after the bPb numbers. Graphical representation of linkage groups and QTL was carried out using MapChart 2.2 [29].

### Statistical analysis

The average values from each experiment were used for the identification of QTL associated with different traits. Using the software package MapQTL6.0 [30], QTL were first analysed by interval mapping (IM). The closest marker at each putative QTL identified using interval mapping was selected as a cofactor and the selected markers were used as genetic background controls in the approximate multiple QTL model (MQM). Logarithm of the odds (LOD) threshold values applied to declare the presence of a QTL were estimated by performing the genome wide permutation tests implemented using at least 1000 permutations of the original data set for each trait, resulting in a 95% LOD threshold around 3.0. After performing restricted MQM mapping which does not use markers close to the QTL, the percentage of variance explained by each QTL (R^2^) was obtained. To detect the effects of plant height on other traits, plant height was chosen as a covariate while conducting QTL analysis for other traits. For the measurements and comparisons of variability among the traits, we calculated the standard deviation (SD). Analysis of variance (ANOVA) and simple correlations among the traits were carried out with the procedures developed by Tang and Feng [31].

## Results

### Genetic linkage map

A high-density genetic linkage map was generated from 75 SSR markers and 551 DArT markers covering a total map distance of 1081.2 cM in seven linkage groups. The average distance between two positions across the whole map was 1.7 cM. The number of markers on different chromosomes ranged from 31 on 6H to 195 on 7H (Figure S1). The marker positions on each chromosome in this map were similar to the published consensus maps [27], [28], [32].

### Traits analysis

The mean values of seven agronomic traits are shown in Table S1. Naso Nijo showed higher values for PH, SL, and AL in all environments while TX9425 had more grain GN and later HD. There were large differences for all traits among DH lines (Table S1). Variance analysis for all traits of the 182 DH lines grown at different environments showed that effects of genotypes, years and the interaction between genotype and year were all highly significant (Table 2).

**Table 2:** Mean squares for agronomic traits of DH lines derived from a Naso Nijo/TX9425 cross.

Source of Variation	HD	PH	SL	AL	GN	KW	GY
**Replication**	**7.09** **	**830.22** **	**1.94** **	**1.7**	**26.67** **	**9.68**	**25856.02** **
**Location(L)**	**1112333.0** **	**76782.33** **	**328.29** **	**55.20** **	**8253.21** **	**4147.72** **	**3368348** **
**Year(Y)**	**513.50** **	**696.40** **	**15.72** **	**0.95**	**423.88** **	**28.51** **	**39194.47** **
**Genotype(G)**	**485.28** **	**296.45** **	**1.40** **	**2.99** **	**29.40** **	**32.51** **	**10277.23** **
**L×Y**	**0.47**	**67.14**	**2.56** **	**0.98**	**20.87** **	**35.3** **	**9537.94** **
**G×L**	**62.04** **	**295.22** **	**0.23** **	**0.67** **	**8.44** **	**53.74** **	**12866.81** **
**G×Y**	**41.28** **	**129.74** **	**0.72** **	**2.10** **	**12.22** **	**28.04** **	**8504.56** **
**G×L×Y**	**9.34** **	**41.48** **	**0.20** **	**0.49**	**3.52** **	**14.75** **	**5618.20** **
**Error**	**1.48**	**20.60**	**0.16**	**0.43**	**2.58**	**3.81**	**1529.36**

** Significant at the 1% level.

Abbreviations for traits are shown in Table 1.

### QTL analysis

Before analysis, DH lines (6 of 188) with possible *uzu1* gene were eliminated by selecting spike morphology similar to Naso Nijo as that semi-dwarfing gene from TX9425 was closely linked with grain density, spike length and awn length [16].

#### QTL for plant height

**Figure 1:**
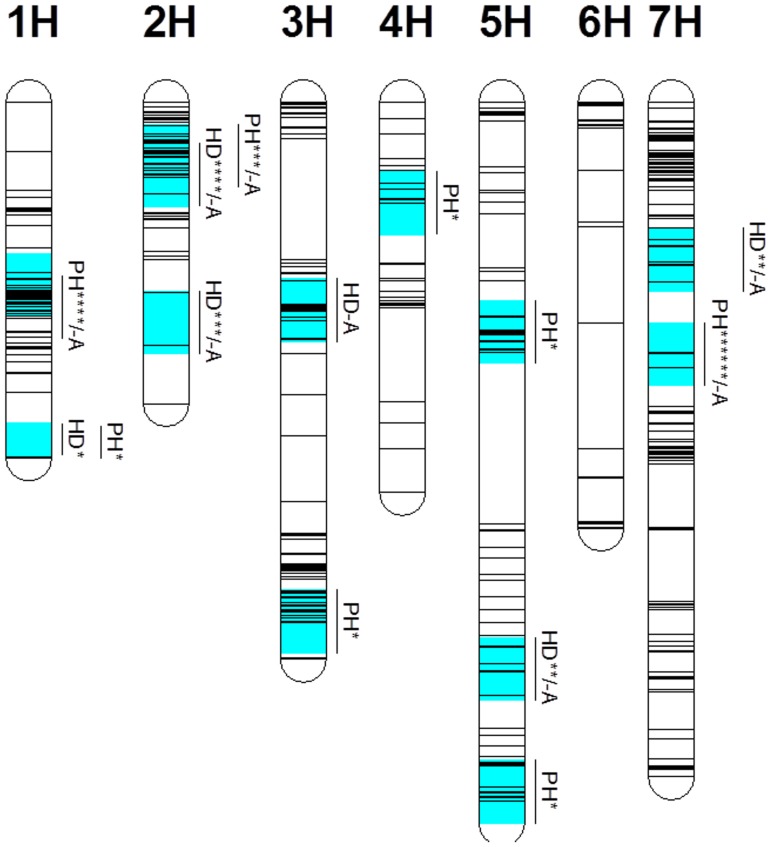
QTL for plant height (PH) and heading date (HD). The number of ‘*’ indicating the number of environments that QTL was detected and ‘-A’ indicating that the QTL was detected based on the average values of all environments. For detail map with markers, see Figure S1.

**Figure 2:**
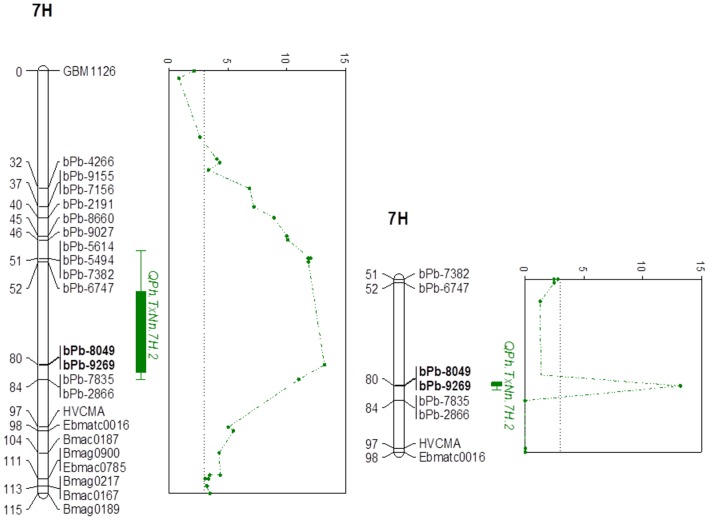
The major QTL on 7H for plant height. Left: rMQM mapping results; right: MQM mapping results. Only a few selected markers were presented. For detailed map, see Figure S1. Markers in bold are the nearest markers to the QTL.

Based on average values from all different environments, three QTL were found to be associated with PH (Figure 1, Table 3). QPh.NaTx-1H explained 10.8% of genetic variance, with bPb-9611 being the closest marker. QPh.NaTx-2H was located on 2H with nearest marker being bPb-6897, explaining 6.7% of genetic variation. A major QTL, QPh.NaTx-7H, was found on 7H with a closest marker of bPb-9269. This QTL explained 23.2% of genetic variance with a LOD value of 13.2 (Figure 2, Table 3). Environments showed significant effects on PH. As shown in Table S2, of all three QTL detected using average values from six environments, QPh.NaTx-1H was not identified in BS and QPh.NaTx-2H was not identified in YC. In contrast, QPh.NaTx-7H was identified in all environments. Figure S2 shows the frequency distribution for plant height of lines with TX9425 and Naso Nijo alleles at the nearest marker bPb-9269. The dwarfing alleles for these three QTL were all from Naso Nijo. Some more QTL were also found from different environments. These included one on 1H from BS07 trial, one on 3H from HZ07 trial, one on 4H from YC06 trial, two QTL on 5H from YC07 trial, and one on 7H from both YC06 and HZ07 trials.

**Table 3:** QTL for agronomic traits and spike morphology detected in the DH population derived from a TX9425/Naso Nijo cross (Average value).

Trait	Linkage group	QTL name	Nearest marker	Position (cM)	LOD	R^2^ (%)	Source of positive effect
PH	**1H**	***QPh.NaTx-1H***	**bPb-9611**	**61.8**	**6.72**	**10.8**	**TX**
	**2H**	***QPh.NaTx-2H***	**bPb-6897**	**17.3**	**4.28**	**6.7**	**TX**
	**7H**	***QPh.NaTx-7H***	**bPb-9269**	**80.0**	**13.23**	**23.2**	**TX**
HD	**1H**	***QHd.NaTx-1H***	**bPb-9108**	**112.6**	**3.68**	**4.8**	**NN**
	**2H**	***QHd.NaTx-2H.1***	**bPb-8292**	**23.1**	**5.68**	**10.6**	**NN**
	**2H**	***QHd.NaTx-2H.2***	**bPb-5449**	**77.4**	**6.47**	**12.0**	**TX**
	**5H**	***QHd.NaTx-5H***	**bPb-2314**	**180.8**	**7.91**	**12.0**	**TX**
	**7H**	***QHd.NaTx-7H***	**bPb-6747**	**57.1**	**6.59**	**9.9**	**NN**
SL	**1H**	***QSl.NaTx-1H***	**bPb-3920**	**80.1**	**4.08**	**5.14**	**NN**
	**2H**	***QSl.NaTx-2H***	**bPb-1196**	**36.2**	**9.78**	**14.0**	**TX**
	**5H**	***QSl.NaTx-5H***	**AWBMS0054**	**79.5**	**14.4**	**21.8**	**TX**
	**7H**	***QSl.NaTx-7H***	**bPb-6747**	**84.2**	**5.10**	**7.0**	**TX**
AL	**1H**	***QAl.NaTx-1H***	**0501C**	**66.0**	**6.67**	**7.3**	**TX**
	**3H**	***QAl.NaTx-3H.1***	**bPb-3282**	**8.2**	**9.08**	**12.5**	**NN**
	**3H**	***QAl.NaTx-3H.2***	**bPb-3634**	**149.0**	**7.39**	**10.4**	**TX**
	**5H**	***QAl.NaTx-5H***	**Bmag0751**	**75.8**	**13.43**	**16.0**	**TX**
	**7H**	***QAl.NaTx-7H.1***	**bPb-7360**	**11.0**	**3.34**	**4.9**	**NN**
	**7H**	***QAl.NaTx-7H.2***	**bPb-8351**	**98.3**	**3.73**	**5.4**	**TX**
	**7H**	***QAl.NaTx-7H.3***	**bPb-8778**	**180.0**	**7.14**	**10.0**	**NN**
GN	**1H**	***QGn.NaTx-1H***	**Bmac0063**	**67.8**	**4.23**	**4.7**	**NN**
	**2H**	***QGn.NaTx-2H***	**bPb-6755**	**48.9**	**5.3**	**5.9**	**TX**
	**3H**	***QGn.NaTx-3H***	**bPb-3634**	**149.0**	**6.49**	**7.41**	**TX**
	**4H**	***QGn.NaTx-4H***	**bPb-0076**	**30.4**	**12.96**	**16.1**	**TX**
	**7H**	***QGn.NaTx-7H.1***	**bPb-7382**	**51.5**	**6.06**	**8.1**	**TX**
	**7H**	***QGn.NaTx-7H.2***	**bPb-6301**	**158.7**	**6.24**	**8.3**	**TX**

The position is that of the nearest marker; R^2^ means percentage genetic variance explained by the nearest marker.

#### QTL for heading date (HD)

Five QTL (*QHd.NaTx-1H, QHd.NaTx-2H.1, QHd.NaTx-2H.2, QHd.NaTx-5H and QHd.NaTx-7H*) were detected for HD based on the average values from all environments (Table 3). These QTL explained 4.8 – 12.0% of genetic variation. The total genetic variation explained by these five QTL was about 50%. However, all the QTL showed significant interactions with environments. *QHd.NaTx-1H* was only detected in BS06 trial and *QHd.NaTx-7H* was only detected in BS trial. None of the QTL was identified in more than three environments.

#### QTL for spike morphology

Four QTL were identified for SL (Table 3). Among these QTL, *QSl.NaTx-1H* was only significant in HZ06 and YC06 trials, *QSl.NaTx-2H* and *QSl.NaTx-7H* was significant in four of six environments, while *QSl.NaTx-5H* was significant in all the environments. TX9425 allele increased SL in most QTL (Table 3). Seven QTL were found for AL, explaining a total of more than 65% of phenotypic variation. TX9425 allele increased AL in four of the QTL. Except for *QAl.NaTx-7H.1* and *QAl.NaTx-7H.2*, all the QTL were significant in three or more environments. Six QTL controlling GN were found, explaining 4.7% – 16.1% of the phenotypic variation (Table 3). TX9425 allele increased GN in five QTL while Naso Nijo allele increased GN in only one QTL.

#### QTL for 1000-kernel weight (KW)

The number of detected QTL for KW varies from one (from HZ06, YC07 and BS07 trial) to five (from HZ07 trial) and different QTL were detected from different environments. Most of the QTL were located in similar positions to those for PH. When PH was added to the analysis as a covariate, three of the five QTL from HZ07 could not be detected and the R^2^ for the major QTL on 3H reduced from 21% to 4%. The QTL for KW from YC06 were also affected by PH with the total R^2^ from 35% to 15% when PH was used as a covariate (Table 4).

**Table 4:** QTL for KW and GY detected in a DH population derived from a TX9425/Naso Nijo cross grown in different environments.

Trait	Linkage group	Nearest marker	Position (cM)	LOD	R^2^ (%)	PH as covariate
**KW**						
HZ06	**3H**	**bPb-7770**	**8.2**	**4.68**	**11.2**	**Unchanged**
YC06	**1H**	**Bmag0211**	**68.7**	**7.85**	**15.2**	**Down**
	**7H**	**HVWAXYG**	**26**	**9.98**	**19.8**	**Down**
BS06	**1H**	**Bmag0347**	**64**	**3.89**	**7.6**	**Unchanged**
	**2H**	**bPb-4740**	**17**	**8.91**	**18.6**	**Unchanged**
HZ07	**1H**	**Bmag0345**	**91**	**4.33**	**6.2**	**X** *
	**2H**	**bPb-8257**	**15**	**5.76**	**8.4**	**X**
	**3H**	**bPb-7770**	**8.2**	**4.02**	**8.0**	**Unchanged**
	**3H**	**bPb-8123**	**163**	**11.66**	**21.1**	**Down**
	**4H**	**GBS0692**	**110**	**3.49**	**4.9**	**X**
YC07	**5H**	**GMS0002**	**218**	**3.0**	**7.1**	**X**
BS07	**2H**	**bPb-4740**	**17.0**	**4.83**	**11.5**	**Unchanged**
**GY**						
HZ06	**2H**	**bPb-5449**	**77.4**	**3.11**	**7.6**	**Unchanged**
YC06	**1H**	**HVM20**	**64**	**5.23**	**8.8**	**X**
	**3H**	**bPb-1799**	**4**	**3.92**	**4.5**	**Unchanged**
	**4H**	**bPb-8169**	**32**	**3.72**	**6.1**	**X**
	**7H**	**bPb-6453**	**24.7**	**9.42**	**16.7**	**X**
BS06	**3H**	**bPb-5523**	**165.3**	**3.47**	**8.4**	**Unchanged**
HZ07	**3H**	**bPb-8123**	**163**	**11.63**	**24**	**Down**
	**5H**	**GMS001**	**21**	**3.07**	**5.7**	**Unchanged**

The position is that of the nearest marker; R^2^ means percentage genetic variance explained by the nearest marker.

*X: No significant QTL was detected.

#### QTL for grain yield per plot (GY)

The QTL identified for GY varies between environments. There was no QTL identified from YC07 and BS07 trials and only one minor QTL was identified from HZ06 and BS06 trials. Two and four QTL were identified from HZ07 and YC06 trials, respectively (Table 4). Three of the four QTL detected from YC06 trial were in similar positions to QTL for PH. When analysing QTL using PH as a covariate, all the three QTL for GY from YC06 trial became insignificant with only one minor QTL on 3H being detected (Table 4). One of the major QTL detected in HZ07 trial was also in a similar position of a major QTL for PH. After adding PH to the analysis, phenotypic variation of GY determined by the QTL reduced from 24% to 11%.

## Discussion

Most agronomic traits are quantitative and QTL mapping for these traits are affected by many factors, including populations, environments, methods for measuring traits, molecular markers and maps. Despite these uncertainties, comparisons among QTL studies can reveal chromosome regions, and provide guidance for the eventual identification of specific genes that are responsible for quantitative trait variation. In this study, a DH population consisting of 182 lines was evaluated for plant height and other agronomic and yield traits in six different environments. Most of the QTL for different traits varied between environments, i.e. a QTL detected in one environment could not necessarily be detected in another environment (Figure 1, Table S2).

Plant height is always one of the most important traits for barley. The use of semi-dwarf genes has greatly improved barley yields with controlled plant height being used to reduce yield loss arising from lodging and to increase the harvest index [14]. Many QTL conferring plant height have been reported and are detected in all 7 chromosomes [33]-[39]. However, most of the QTL determined only a small amount of phenotypic variation and can be easily affected by environment. For example, Pillen et al. [38] found that plant height was controlled by more than 10 QTL, which makes it hard for plant breeders to use molecular markers to select for this trait. Thus, to be effective for MAS, the QTL should have sufficient allelic effect and express in a known environment. Several major semi-dwarf genes were identified and widely used in breeding programs. These genes include *uzu1* [5], *sdw1* or *denso* [6], *ari-e* [7] and *hcm1* [8]. In our previous studies, two dwarf genes were identified from a Chinese landrace variety TX9425 [10]. One of the dwarf genes was located at a same position of *uzu1*, which also had pleiotropic effects on some unfavourable spike morphology, including grain density, spike length and awn length [16]. The current study was to identify any new dwarfing genes outside the region where *uzu1* is located and whether the dwarfing gene is from TX9425 or the other parent variety, Naso Nijo. For this purpose, six lines with TX9425 type of spike morphology (short awn and twist spike) were excluded from further analysis. A total of nine QTL for plant height were identified. However, most of them either had small allelic effect or was only detected in a single environment, which are less useful for MAS. Two QTL (*QPh.NaTx-1H* and *QPh.NaTx-2H*) were identified in more than three environments and determined 10.8% and 6.7% of phenotypic variation, respectively, on the bases of average values from six environments (Figure 1, Table 3). These two QTL were located on similar positions according to previously reported [34]–[36]. Both can possibly be used for MAS. The most important one on 7H (*QPh.NaTx-7H*) was consistently expressed in all of the environments and determined 23% of phenotypic variation (Figure 2, Table 3). Several studies also reported QTL on 7H but these QTL were either located at different positions of the chromosome or no obvious evidence of a major gene [13], [14], [36]–[42]. Qi et al [43] identified a major QTL for plant height on 7H and the position is similar to the one identified in this experiment. However, the QTL reported by them showed a close linkage with heading date while the QTL identified from this population showed no relationship with heading date. Sameri et al. [44] reported a QTL on chromosome 7H for reduced culm internode length from the Japanese variety Kanto Nakate Gold. This QTL is in a similar position to a major semi-dwarfing gene reported by Yu et al. [13]. However, the position of the QTL (or the semi-dwarfing gene) is different from the QTL identified in this study, which is nearly 100 cM away by locating both QTL on the consensus maps [27], [28], [32], [45].

**Figure 3:**
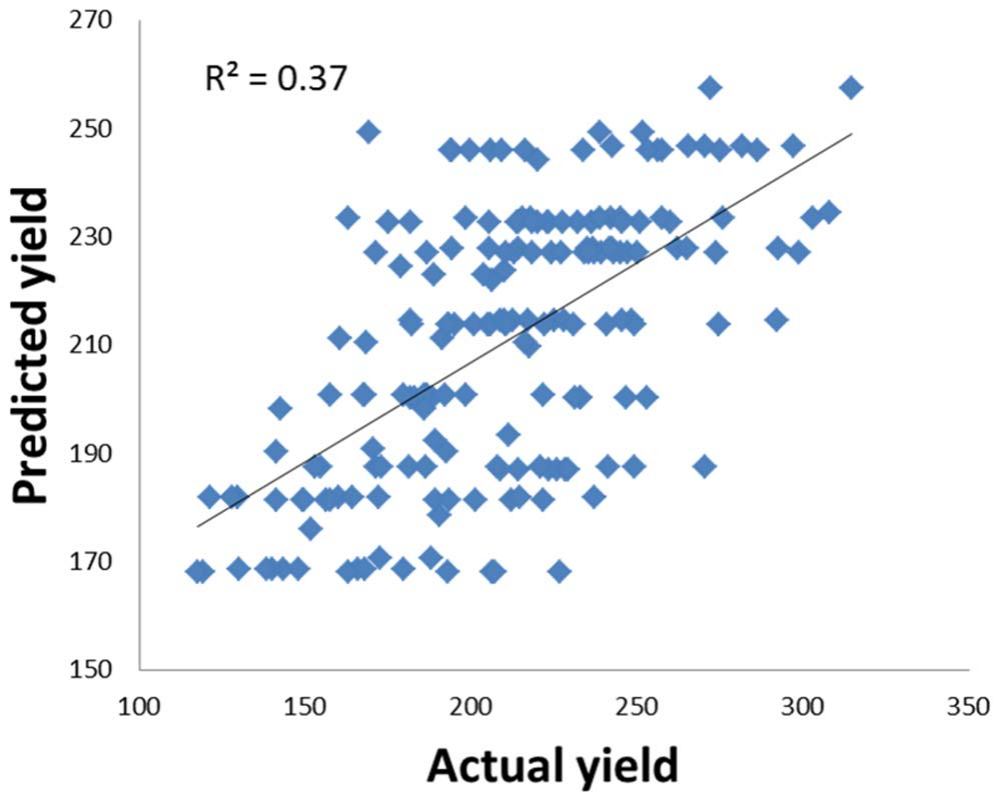
The correlation between actual grain yields and yields predicted using makers linked to QTL for PH on 1H (QPh.NaTx-1H), 2H (QPh.NaTx-2H), 3H(QPh3H) and 7H (QPh7H) (Table S1).

Many semi-dwarfing genes have some problems in their use in breeding programs. Both *uzu1* and *sdw1* showed some undesirable pleiotropic effects on other traits [10], [16], [17]. QTL identified in this study showed little effect on spike morphology even though some QTL were located on the similar positions to those for spike morphology. Some of the previously reported dwarfing genes also have pleiotropic effects on heading dates with *sdw1* tending to delay heading and *uzu1* being temperature and/or day length sensitive. In this study, five QTL were identified for heading date and were located on chromosomes 1H, 2H, 5H and 7H. When using PH as a covariate, most QTL for HD were still detected, indicating the weak linkage between PH and HD in this cross. However, most of the QTL for PH showed significant effects on GY and KW. For examples, when QTL analysis for GY was conducted using PH as a covariate, the QTL on 1H for GY detected from YC06 trial and QTL on 1H and 2H for KW detected from HZ07 trial became insignificant. As shown in Figure 1 and Table S2, several other QTL for PH were identified in one of six environments. These QTL not only showed environmental-dependency but also a great effect on KW and GY. When PH was added to the analysis as a covariate, the R^2^ for the major QTL for KW on 3H detected from HZ07 trial (which is located on a similar position to the QTL for PH) reduced from 21% to 4% (Table 4). No QTL for either GY or KW were detected in the region where the other major QTL for PH on 7H (*QPh.NaTx-7H*) was located (Tables 3, 4). To further confirm that the QTL on 7H for PH had no effect on yield, a regression analysis was conducted using the data from HZ07 where several QTL were found for GY. The nearest markers for different QTL for PH were selected to conduct regression analysis to GY. Results showed that only the marker linked to *QPh.NaTx-7H* had no significant contribution to GY while markers linked to other four QTL contributed a total of 37% of GY variation (Figure 3).

In conclusion, a new QTL for plant height was identified on 7H. Unlike most of other dwarf genes, this gene showed no significant effects on other agronomic traits and yield components. This QTL also showed a sufficient allelic effect and consistently expressed in various environments and thus can be successfully used in breeding programs.

## Supporting Information

Figure S1 Barley genetic linkage map of Naso Nijo/TX9425 population based on DArT and SSR markers.
(DOCX)

Figure S2 Frequency distribution for plant height of lines with TX9425 and Naso Nijo alleles at the nearest marker bPb-9269.
(DOCX)

Table S1 Mean and range of agronomic traits tested in six environments.
(DOCX)

Table S2 QTLs for plant height detected in different environments.
(DOCX)
