# Supplementary material for: A New QTL for Plant Height in Barley (Hordeum vulgare L.) Showing No Negative Effects on Grain Yield
Source: PLoS One. 2014 Feb 28;9(2):e90144. doi: 10.1371/journal.pone.0090144 (PMC3938599; doi:10.1371/journal.pone.0090144)
Supplement: Figure S1 — Barley genetic linkage map of Naso Nijo/TX9425 population based on DArT and SSR markers. (DOCX) [file pone.0090144.s001.docx]

**Figure S1. Barley genetic linkage map of Naso Nijo/TX9425 population based on DArT and SSR markers.**
