# Supplementary material for: A New QTL for Plant Height in Barley (Hordeum vulgare L.) Showing No Negative Effects on Grain Yield
Source: PLoS One. 2014 Feb 28;9(2):e90144. doi: 10.1371/journal.pone.0090144 (PMC3938599; doi:10.1371/journal.pone.0090144)
Supplement: Figure S2 — Frequency distribution for plant height of lines with TX9425 and Naso Nijo alleles at the nearest marker bPb-9269. (DOCX) [file pone.0090144.s002.docx]

Naso Nijo allele

TX9425 allele

**Figure S2. Frequency distribution for plant height of lines with TX9425 and Naso Nijo alleles at the nearest marker bPb-9269.**
