# Supplementary material for: A New QTL for Plant Height in Barley (Hordeum vulgare L.) Showing No Negative Effects on Grain Yield
Source: PLoS One. 2014 Feb 28;9(2):e90144. doi: 10.1371/journal.pone.0090144 (PMC3938599; doi:10.1371/journal.pone.0090144)
Supplement: Table S1 — Mean and range of agronomic traits tested in six environments. (DOCX) [file pone.0090144.s003.docx]

**Table S1 Mean and range of agronomic traits tested in six environments**

| **Trait** | **Environment** | **TX9425** | **Naso Nijo** | DH lines | |
| --- | --- | --- | --- | --- | --- |
|  |  |  |  | **Mean****±SD** | **Range** |
| **HD** | HZ07 | 123 | 116.5 | 117 ± 2.85 | 112-124 |
|  | HZ08 | 134 | 132 | 132 ± 1.77 | 129-135 |
|  | YC07 | 151.5 | 149.5 | 148 ± 2.55 | 141-156 |
|  | YC08 | 154 | 151 | 151 ± 1.90 | 147-155 |
|  | BS07 | 88 | 81.5 | 80 ± 3.57 | 72-89 |
|  | BS08 | 96 | 88 | 92 ± 2.76 | 85-98 |
| **PH** | HZ07 | 99.6 | 103.8 | 107 ± 4.27 | 97-120 |
|  | HZ08 | 97 | 100 | 104 **±** 5.73 | 86-119 |
|  | YC07 | 86 | 87 | 82 ± 6.15 | 58-98 |
|  | YC08 | 95 | 98 | 102 ± 3.35 | 92-113 |
|  | BS07 | 85.4 | 89.7 | 91 ± 5.4 | 80-107 |
|  | BS08 | 77 | 84 | 91 ± 5.7 | 73-104 |
| **SL** | HZ07 | 5.5 | 6.3 | 6.4 ± 0.34 | 5.6-7.2 |
|  | HZ08 | 5.6 | 6.5 | 6.9 ± 0.38 | 5.8-7.9 |
|  | YC07 | 6.1 | 6.8 | 6.7 ± 0.46 | 5.6-8.4 |
|  | YC08 | 5.8 | 6.8 | 6.9 ± 0.43 | 5.9-8.3 |
|  | BS07 | 5.2 | 5.7 | 5.7 ± 0.34 | 4.9-6.5 |
|  | BS08 | 5.3 | 5.6 | 5.9 ± 0.34 | 5.0-7.4 |
| **AL** | HZ07 | 5.5 | 11.2 | 11.1 ± 0.64 | 9.5-12.9 |
|  | HZ08 | 5.0 | 10.7 | 11.1 ± 0.61 | 9.2-13.1 |
|  | YC07 | 5.7 | 11.8 | 11.6 ± 0.64 | 10.2-13.2 |
|  | YC08 | 5.3 | 11.2 | 11.4 ± 0.0.63 | 9.5-13.1 |
|  | BS07 | 5.8 | 11.3 | 10.9± 0.63 | 9.1-12.4 |
|  | BS08 | 5.7 | 11.4 | 11.3 ± 0.62 | 9.6-12.7 |
| **GN** | HZ07 | 24.6 | 22.7 | 25.5 ± 1.2 | 22.8-28.6 |
|  | HZ08 | 31.1 | 27.3 | 29.7 ± 1.27 | 26.4-32.7 |
|  | YC07 | 34.9 | 28.1 | 27.6 ± 1.73 | 23.7-33.5 |
|  | YC08 | 31.1 | 28.1 | 28.8 ± 1.57 | 24.1-33.4 |
|  | BS07 | 24.2 | 20.0 | 22.9 ± 1.38 | 18.1-26.6 |
|  | BS08 | 23.6 | 20.2 | 23.5 ± 1.51 | 19.9-28.2 |
| **KW** | HZ07 | 37.9 | 38.1 | 37.3 ± 2.02 | 31.9-42.2 |
|  | HZ08 | 32.4 | 33.1 | 33.9 ± 2.78 | 26.5-40.4 |
|  | YC07 | 37.4 | 36.4 | 34.8 ± 3.76 | 27.1-43.7 |
|  | YC08 | 38.9 | 38.8 | 40.0 ± 2.61 | 34.2-49.0 |
|  | BS07 | 37.9 | 37.6 | 41.5 ± 2.59 | 34.5-49.5 |
|  | BS08 | 35.8 | 35.9 | 37.5 ± 1.68 | 33.3-41.7 |
| **GY** | HZ07 | 241.2 | 342.2 | 307.4 ± 42.57 | 189-444 |
|  | HZ08 | 162.9 | 236.1 | 213.0±39.15 | 120-315 |
|  | YC07 | 297.2 | 356.7 | 287.9±67.07 | 122-453 |
|  | YC08 | 350.8 | 368.7 | 322.3±53.59 | 187-501 |
|  | BS07 | 169.4 | 216.4 | 189.7± 39.48 | 63-291 |
|  | BS08 | 160.0 | 198.5 | 199.7±36.04 | 80-303 |

SD means standard deviation. Abbreviations for traits are shown in Table 1
