# Supplementary material for: A New QTL for Plant Height in Barley (Hordeum vulgare L.) Showing No Negative Effects on Grain Yield
Source: PLoS One. 2014 Feb 28;9(2):e90144. doi: 10.1371/journal.pone.0090144 (PMC3938599; doi:10.1371/journal.pone.0090144)
Supplement: Table S2 — QTLs for plant height detected in different environments. (DOCX) [file pone.0090144.s004.docx]

**Table S2 QTLs for plant height detected in different environments**

| **QTL** | | **Chro.** | | **Position**  **(cM)** | | **Environment** | | | | | | | | | | | | **Av** | |
| --- | --- | --- | --- | --- | --- | --- | --- | --- | --- | --- | --- | --- | --- | --- | --- | --- | --- | --- | --- |
|  |  |  |  |  |  | **HZ06** | | **YC06** | | **BS06** | | **HZ07** | | **YC07** | | **BS07** | |  |  |
| ***QPh.NaTx-1H*** | 1H | | 62 | | √ | | **√** | |  | | **√** | | **√** | |  | | **√** | |  |
| ***QPh.NaTx-2H*** | 2H | | 17 | | √ | |  | | **√** | | **√** | |  | |  | | **√** | |  |
| ***QPh.NaTx-7H*** | 7H | | 80 | | √ | | **√** | | **√** | | **√** | | **√** | | **√** | | **√** | |  |
| **QPh1H** | 1H | | 113 | |  | |  | |  | |  | |  | | **√** | |  | |  |
| **QPh3H** | 3H | | 163 | |  | |  | |  | | **√** | |  | |  | |  | |  |
| **QPh4H** | 4H | | 32 | |  | | **√** | |  | |  | |  | |  | |  | |  |
| **QPh5H.1** | 5H | | 73 | |  | |  | |  | |  | | **√** | |  | |  | |  |
| **QPh5H.2** | 5H | | 218 | |  | |  | |  | |  | | **√** | |  | |  | |  |
| **QPh7H** | 7H | | 21 | |  | | **√** | |  | | **√** | |  | |  | |  | |  |
